# The Motor Neuron Disease Register for England, Wales, and Northern Ireland: Protocol for a Population Register

**DOI:** 10.2196/86458

**Published:** 2026-06-03

**Authors:** Andrea Biondi, Elizabeth Gray, Modupe Aggreh, Edward Jones, Amber Collingwood, Liam Knox, Andrea Bredin, David Setters, Kevin Talbot, Ammar Al-Chalabi

**Affiliations:** 1 Department of Basic and Clinical Neuroscience, Maurice Wohl Clinical Neuroscience Institute, Institute of Psychiatry, Psychology and Neuroscience, King's College London, London, England, SE5 9RT, United Kingdom, 44 20 7848 0259; 2 Kavli Institute for Nanoscience Discovery, University of Oxford, Oxford, England, United Kingdom; 3 Sheffield Institute for Translational Neuroscience (SITraN), University of Sheffield, Sheffield, England, United Kingdom; 4 Dorothy Crowfoot Hodgkin Building, Kavli Institute for Nanoscience Discovery, University of Oxford, Oxford, England, United Kingdom; 5 John Radcliffe Hospital, Oxford Motor Neuron Disease Centre, Nuffield Department of Clinical Neurosciences, University of Oxford, Oxford, England, United Kingdom

**Keywords:** protocol, amyotrophic lateral sclerosis, motor neuron disease, national register, methodology

## Abstract

**Background:**

Despite the existence of several regional registries in the United Kingdom, gaps in geographic coverage have limited the ability to produce accurate national estimates of incidence, prevalence, and regional variation for motor neuron disease (MND). To address these challenges, a comprehensive national register encompassing England, Wales, and Northern Ireland was established to support epidemiological studies, health care planning, and clinical research.

**Objective:**

The primary objective of the MND Register is to provide a centralized research database aggregating clinical and demographic data to facilitate high-quality research. Secondary objectives include estimating disease incidence and prevalence, identifying regional differences in care and survival, evaluating potential disease clustering, and supporting data linkage and clinical trial recruitment.

**Methods:**

Eligible patients are those aged 16 years or older with a confirmed MND diagnosis made by a consultant neurologist. Data are collected prospectively and retrospectively through standardized templates, available via Microsoft Access, Microsoft Excel, or the REDCap (Research Electronic Data Capture; Vanderbilt University) web platform, and include up to 34 demographic and clinical variables. Additional self-reported data can be contributed through the Telehealth in MND–Research platform. All data are securely stored in the King’s College London Trusted Research Environment, undergo standardized preprocessing, and may be linked to National Health Service and national datasets for epidemiological analyses.

**Results:**

The register includes data on over 11,000 individuals with MND, of whom nearly 7000 are currently alive. Postcode data are available for more than 4300 patients, enabling future geospatial analyses. By October 2025, 60 clinical sites were participating in the register, with around 50 actively submitting data.

**Conclusions:**

The MND Register represents one of the largest national registries for MND worldwide, providing a robust foundation for epidemiological modeling, clinical research, and health care planning. Ongoing efforts to expand prospective data collection, improve completeness, and integrate digital tools will further enhance its impact and support national and international MND research collaborations.

## Introduction

### Background and Rationale

Motor neuron disease (MND), also known as amyotrophic lateral sclerosis (ALS), is a progressive neurodegenerative disorder characterized primarily by the degeneration of motor neurons, leading to muscle weakness, paralysis, and ultimately respiratory failure, resulting in death in a median of 2 years from diagnosis. The disease has a lifetime risk of approximately 1 in 300, with a median age of onset of 64 years [1]. In about 10% of people, a family history of ALS is identified. The heritability of ALS is about 40% to 60% [2,3], and about 20% of people without a family history have an actionable genetic variant [4]. Additionally, MND shares pathological and genetic features with other neurodegenerative diseases, including frontotemporal dementia, schizophrenia, and Parkinson disease [5].

In the United Kingdom, the incidence of MND has been increasing due to an aging demographic, with the number of cases projected to rise from 1415 in 2010 to 1701 by 2020, and further to 2635 by 2116 [6]. Disease registries play a crucial role in enhancing understanding of the natural history of MND, characterizing its phenotypic variations, and providing insights into prognosis. Registries also facilitate the development of clinical assessment tools and inform health care planning [7,8].

Despite the existence of 5 regional population-based MND registers in the United Kingdom, including the South-East ALS Register, the Peninsula Network, the South Wales Register, the Northern Ireland Register, and MND Care in Scotland—now incorporated into the national MND Register—these registers were previously separate and left gaps in national coverage [9]. As a result, accurate estimates of MND prevalence and distribution across the United Kingdom remained uncertain.

A national MND register encompassing England, Wales, and Northern Ireland is essential for capturing a comprehensive dataset that can accurately determine disease incidence, monitor regional variations in survival rates, and evaluate health care accessibility. Such a resource would allow for advanced epidemiological modeling, aiding in drug discovery efforts and improving overall understanding of MND pathogenesis [10,11]. The collection of nationwide data is also critical for identifying potential disease clusters and ensuring equitable health care resource distribution [8].

Several countries have implemented national ALS registries to facilitate disease surveillance, research collaboration, and clinical trial recruitment. For instance, the National ALS Registry in the United States, established in 2010 by the Centers for Disease Control and Prevention, integrates administrative health records with patient self-enrollment to track ALS incidence and prevalence [4]. Similarly, the Canadian Neuromuscular Disease Registry collects ALS data alongside other neuromuscular disorders to support diagnostic accuracy and research participation [12]. In Sweden, the Swedish Quality Register for MND, launched in 2015, standardizes data collection at regular intervals, incorporating patient-reported outcomes to improve clinical care and research efforts [13]. Other established registries, such as the Italian Neuromuscular Disease Registry [14] and the New Zealand Motor Neurone Disease Registry [15], have also integrated their data collection processes into international collaborations, fostering cross-border research initiatives.

Recognizing the necessity for a comprehensive national dataset, the MND Register for England, Wales, and Northern Ireland was established. Scotland operates a separate national MND registry and is, therefore, not included in this database [16]. Originally developed in London in the 1990s, this initiative has expanded into a nationwide project designed to systematically collect demographic, clinical, and epidemiological data from individuals diagnosed with MND. The register aims to provide reliable estimates of disease incidence and prevalence, monitor regional disparities in survival and health care access, and support epidemiological research and clinical trial recruitment. By establishing a robust national dataset, the register will play a crucial role in advancing MND research, informing health care policies, and ultimately improving patient outcomes.

By detailing the protocol and methodology of the MND Register for England, Wales, and Northern Ireland, this document aims to facilitate future national and international registry development efforts and contribute to global MND research advancements.

### Primary Objective

The MND research database serves as a central repository of information collected from patients across England, Wales, and Northern Ireland. Its primary function is to facilitate research by providing deidentified data to researchers and collaborators for future studies, thereby supporting advances in understanding and treating MND.

### Secondary Objectives

This dataset will enable the calculation of MND incidence and prevalence across the regions covered. Additionally, the data will be linked with National Health Service (NHS) records, Hospital Episode Statistics, and Office for National Statistics data to investigate regional variations in survival rates and health care usage. The collected information will contribute to the evaluation of regional differences in care planning and service provision. Furthermore, the dataset will facilitate the identification of potential disease clusters and improve the generalizability of MND research findings. By pooling data from multiple research studies, the database will support the investigation of complex research questions and enable the quantification of research participation among patients with MND.

## Methods

### Patient Identification

**Figure 1.**
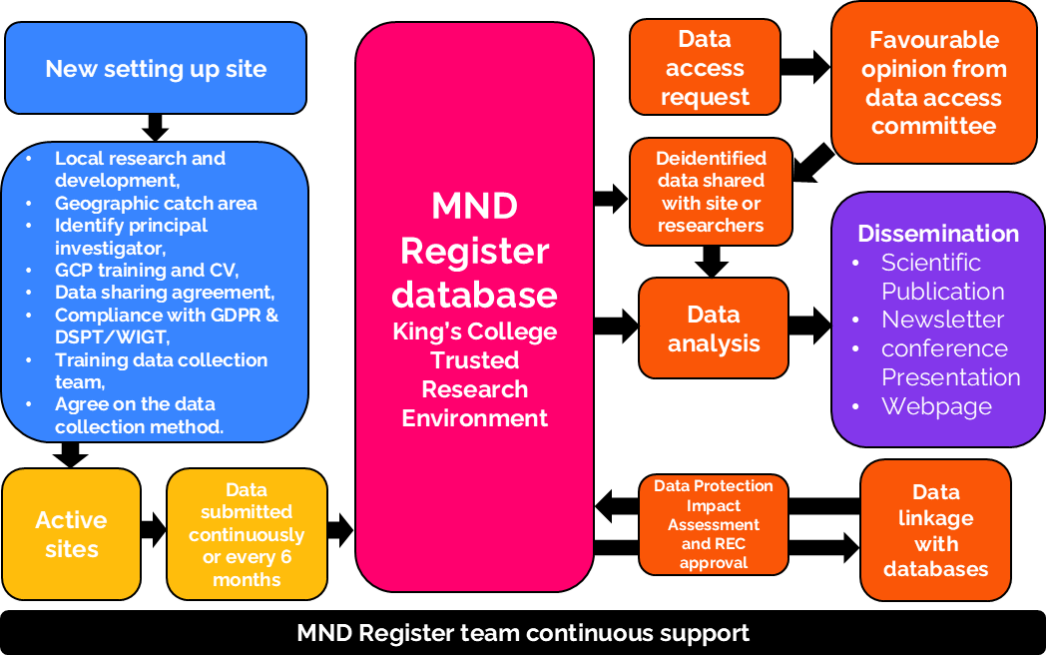
Motor Neuron Disease (MND) Register procedure and structure for sites. From left to right: new sites joining the MND Register will complete the inclusion procedure (blue) and, following all required approvals, will become active sites contributing data on a regular basis (yellow). Submitted data will be incorporated into the MND Register database (purple) and subsequently used for data sharing with other research teams, data analysis, and data linkage, subject to research ethics committee (REC) approval (orange). The resulting findings will be disseminated regularly through scientific publications, conferences, newsletters, and the MND Register website (violet). To ensure the integrity and transparency of the process, the MND Register team will coordinate, support, and monitor participating sites, with all steps conducted in accordance with established governance and data management protocols (black). CV: curriculum vitae; GCP: Good Clinical Practice; GDPR: General Data Protection Regulation; DSPT: Data Protection Security Toolkit; WIGT: Welsh Information Governance Toolkit.

Patients eligible for inclusion in the MND Register are those diagnosed with MND by a consultant neurologist in England, Wales, or Northern Ireland. Figure 1 summarizes the MND Register structure. Identification occurs during routine clinical appointments. Inclusion criteria require a confirmed diagnosis, an age of at least 16 years, and residency in 1 of the 3 participating regions. Patients who do not meet these criteria, including those under 16 years of age or those residing outside the covered regions, are excluded. Participation in the MND Register does not impose any additional costs on patients, as data collection is integrated into routine clinical visits. No financial compensation is provided for participation in the project. The study complies with the General Data Protection Regulation (GDPR) and the Data Protection Act 2018, which require data to be deidentified as soon as it is practical to do so.

### Data Collection and Entry Procedure (for Site)

The MND Register collects data both cross-sectionally and longitudinally from participating hospitals and neurology services using a structured questionnaire. The dataset includes basic demographic details, clinical variables, and GDPR-defined special category data, such as genetic and health information [17]. Participating sites are asked to contribute up to 34 variables, with a minimum dataset consisting of 11 core variables also available (Table 1). A full and detailed data dictionary is available for researchers and collaborators (Multimedia Appendix 1).

**Table 1.** List of the items included in the comprehensive Motor Neuron Disease (MND) Register database. The minimum database includes only a subgroup of 34 items.

No.	Data item	Minimum database	Options or data definition
1	Unique ID number	✓	Automatically assigned
2	First name	✓	
3	Surname	✓	
4	NHS^a^ number	✓	
5	Patient or control		Not included anymore after Section 251 (January 2020)
6	Date of birth	✓	
7	Date of death	✓	
8	Notes		
9	Sex	✓	Assigned at birth
10	Ethnicity		White: (A) British, (B) Irish, and (C) any other White background Mixed: (D) White and Black Caribbean, (E) White and Black African, (F) White and Asian, and (G) any other mixed background Asian or Asian British: (H) Indian, (J) Pakistani, (K) Bangladeshi, and (L) any other Asian background Black or Black British: (M) Caribbean, (N) African, and (P) any other Black background Other ethnic groups: (R) Chinese, (S) any other ethnic group, and (Z) not stated National code Z: Not stated should be used where the PERSON has been given the opportunity to state their ETHNIC CATEGORY but chose not to
11	Postcode of residence at diagnosis	✓	Full post code data collected; for example, SE5 9RT
12	Source of ascertainment		Options include: MND care and research center, specialist neurology outpatient clinic, general neurology outpatient clinic, clinical nurse specialist, community multidisciplinary team, hospice, and other
13	Date of diagnosis	✓	Date that the diagnosis was first discussed with patient by the ascertaining center
14	Diagnosis	✓	Type of MND
15	Comorbidity		Available options in dropdown menus: cancer, cardiovascular disease, Parkinson disease, psychiatric disorder(s), autoimmune disorder(s), and absent or unknown Free text box available for: comorbidity, other Two comorbidities can be indicated
16	Date of symptom onset	✓	Date
17	Site of onset	✓	Site of onset: bulbar, spinal, thoracic or respiratory, generalized (eg, both swallowing issues and weakness in leg reported at the same time) If spinal, then select: arms, legs (dropdown box); and select left, right (dropdown box) Other first symptoms: weight loss (tick box if yes; >10% weight loss in 6 months prior to the time of disease onset as defined by date of onset symptoms) Cognitive or behavioral (tick box if yes)
18	Limb dominance		Leg (left/right) and arm (l/R^b^)
19	Current number of regions affected		Speech, swallowing, hand (left/right), arm (left/right), foot (left/right), leg (left/right) trunk, and breathing
20	Cognitive impairment		Yes or no (and details of formal assessment where applicable)
21	El Escorial category		El Escorial categories: suspected ALS^c^, possible ALS, probable ALS, and definite ALS
22	El Escorial date		Date
23	ALS-FRS-R^d^		Date of ALS-FRS-R (date field); ALS-FRS-R 12 subscores (number fields); ALS-FRS-R total score. Following the validated scale [18]
24	FVC^e^		FVC table (preferred): Date FVC (date field) FVC (open text field) FVC (% of predicted; open text field) Testing not performed or not feasible: not tested, not feasible
25	SNIP^f^		SNIP table (optional) Date SNIP (date field) SNIP (cm H_2_O; open text field) Testing not performed or not feasible: not tested, not feasible
26	Family history		Family history: number of affected family members with ALS or MND and FTD^g^ or dementia NIP^h^ table (optional): Date SNIP (date field) SNIP (cm H_2_O; open text field) Testing not performed or not feasible: not tested, not feasible
27	Progression	✓ (only for date of death if available)	Date of death (date field) If tracheostomy (yes or no), date of tracheostomy (date field) If>23 hours NIV^i^/day, date of end point (date field) Date of checking (if not deceased, no tracheostomy and no>23 hours NIV/day; date field)
28	ECAS^j^ cognitive screen		Following validated ECAS form [19]
29	ECAS behavioral screen		Following validated ECAS form [19]
30	Height (cm)		
31	Weight (kg)		In kilograms, also ask to report if weight lost (yes vs no)
32	Medications		Yes or no and date
33	Gastrostomy		Yes or no and date
34	Respiratory intervention		Yes or no and date

a NHS: National Health Service.

b I/R: internal rotation.

c ALS: amyotrophic lateral sclerosis.

d ALS-FRS-R: Amyotrophic Lateral Sclerosis Functional Rating Scale–Revised.

e FVC: forced vital capacity.

f SNIP: sniff nasal inspiratory pressure.

g FTD: frontotemporal dementia.

h NIP: nasal inspiratory pressure.

i NIV: noninvasive ventilation.

j ECAS: Edinburgh Cognitive and Behavioural ALS Screen.

During routine clinical appointments, a designated health care team member extracts patient data from medical records according to the MND Register Comprehensive Dataset. This information is entered into the designated template database or, for centers with an existing database, recorded within their system. If patients are unable to attend clinic appointments, notification flyers may be provided in community settings or during home visits for palliative care. These flyers, available in clinics and online through the MND Register website, explain the study and outline the opt-out process. For patients lacking capacity, caregivers or family members may review the notification flyer on their behalf.

Data are collected using one of the following data entry options, depending on site preferences and technical capabilities:

- Offline secure application (Microsoft Access System [20]; comprehensive database): for sites using the complete MND Register database.
- Offline spreadsheet (minimum database): for sites preferring a simplified approach with a minimum dataset.
- Online secure web application (REDCap [Research Electronic Data Capture; Vanderbilt University]; comprehensive database): an alternative to Microsoft Access, offering full database functionality for sites that prefer an online, user-friendly system.

**Figure 2.**
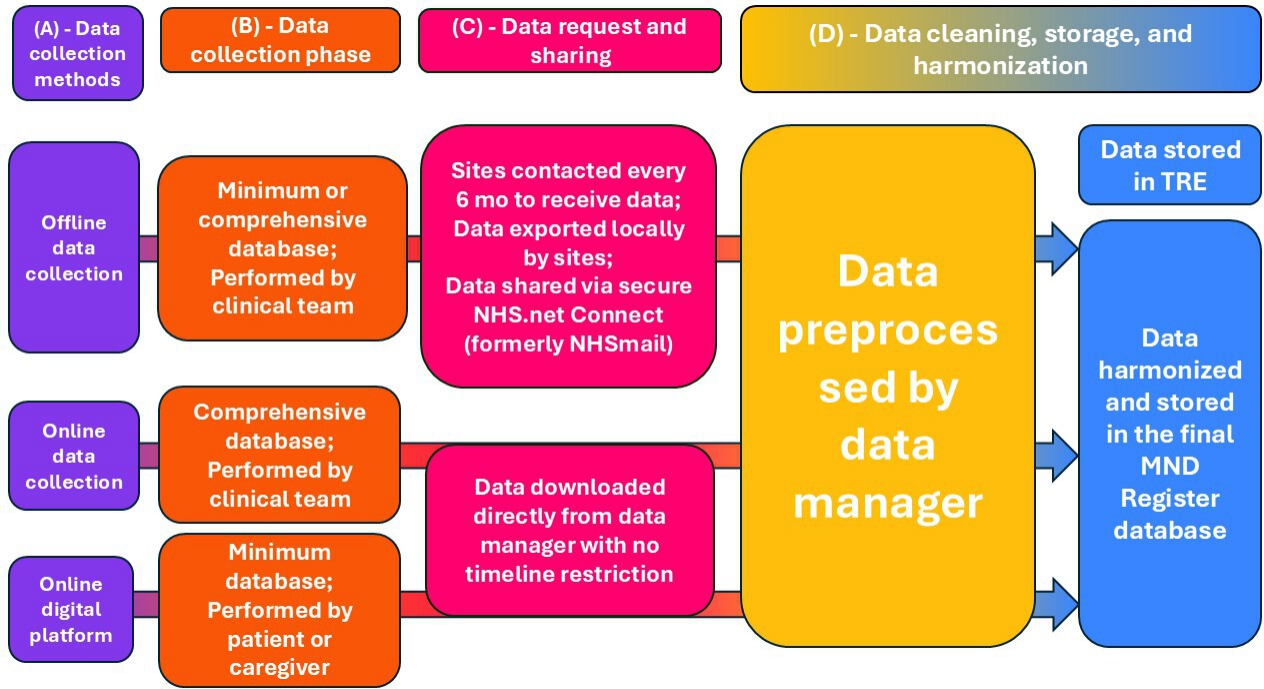
Motor Neuron Disease (MND) Register data flowchart. From left to right: data collection methods (purple) capture a range of variables during the data acquisition phase (orange). The collected data are subsequently requested and transferred to the MND Register teams in Oxford or London (purple). All data are preprocessed at King’s College London (yellow) and then harmonized to create the final MND Register database, which is securely stored within a Trusted Research Environment (TRE; blue). NHS: National Health Service.

Data are then securely submitted via NHS.net email (for methods 1 and 2) to the data scientist at King’s College London (KCL) for cleaning and analysis. The use of a secure web application such as REDCap [21] will be hosted on secure servers at the University of Oxford, ensuring data security with encryption at rest and in transit. Each site using REDCap will receive a single data entry account, managed by the principal investigator, who supervises access and ensures that only trained personnel use the system. Data are entered directly into REDCap following the MND Register Comprehensive Dataset. Data are not transmitted by email but are securely exported to the KCL Trusted Research Environment (TRE) for integration into the central MND Register. Sites using MS Access or spreadsheets transfer identifiable data via encrypted NHS.net email. Detailed data flow for all methods is illustrated in Figure 2.

### Data Collection and Entry Procedure (for Patients and Caregivers)

To facilitate broader participation in the MND Register and ensure alignment with remote research initiatives, patients will be allowed to self-report and include their data (minimum database) in the register using the Telehealth in Motor Neuron Disease–Research (TiM-R) platform [22]. TiM-R is an innovative digital platform developed by the UK MND Research Institute in collaboration with the University of Sheffield. It allows people living with MND to participate in research studies remotely, reducing the burden of travel and enabling more comprehensive and representative data collection. Participants who register with TiM-R will be provided access to the MND Register Notification Flyer and details of the opt-out procedures via the TiM-R platform prior to sharing their data. This process ensures that participants make an informed decision regarding the linkage of their TiM-R data with the MND Register and affirms their opportunity to decline inclusion of their data in the register.

### Data Processing and Harmonization

Data submitted from participating sites may originate from different local data entry systems, including Microsoft Access databases, Microsoft Excel templates, REDCap, or patient-reported data through the TiM-R platform. To ensure consistency across these sources, all datasets undergo centralized preprocessing and harmonization prior to integration into the MND Register database. Data are processed through a structured pipeline developed using Python (Python Software Foundation), R (R Foundation for Statistical Computing), and SQL (Structured Query Language)-based tools (Oracle), which standardize variable names, coding schemes, and formats according to the Register data dictionary. The pipeline includes sequential steps of data ingestion, validation checks, cleaning of inconsistent entries (eg, implausible dates or formatting errors), harmonization of variables across source systems, and deduplication before inclusion in the final database. The progressive adoption of REDCap as an online data collection platform is expected to further improve standardization and streamline data submission across participating centers.

### Data Storage and Security

The MND Register database is securely stored within the KCL CREATE (Computational Research, Engineering and Technology Environment) TRE. Data that are no longer being processed are retained according to the MND Register retention policy and subsequently deleted by the KCL IT team when no longer required. For each participant, a unique ID is created and stored in the central MND Register database. This unique ID is not shared outside of the main MND Research team (data scientists and project managers). When sharing data with other researchers as part of a data access request, we create a separate patient ID to ensure researchers cannot link the data back to the original dataset. Patients retain the right to opt out of data inclusion at any time. Upon opting out, no further work is conducted on their data, although anonymized datasets already in use for research cannot be removed. Opting out does not affect the standard of care received.

### Plan for Data Analysis

Data preprocessing, including the assessment of missing data, quality control, and deduplication, is performed using standardized methods, with the cleaning protocol available upon request for reproducibility.

Duplicate records are identified through deterministic and probabilistic matching using combinations of identifiers such as NHS number, date of birth, and postcode.

Implausible values and inconsistent entries (eg, invalid dates or formatting errors) are flagged during validation checks and reviewed before inclusion in the analytical dataset.

Patterns of missing data will be assessed across variables, and analyses will primarily use available-case approaches, with sensitivity analyses considered where missingness might influence key outcomes.

Descriptive analysis is performed on the database to provide information regarding demographic and clinical characteristics (ie, gender, diagnosis, age of onset, death, survival rates, and interventions) and any other factors that may affect outcomes. Age and sex-adjusted incidence rates are calculated by categorizing patients by age and sex in 10-year bins within defined catchment areas. Population data are used to estimate person-years of observation and to adjust incidence rates accordingly. Point and period prevalences will be estimated for each region. Geospatial analysis using postcode data will facilitate the mapping of MND distribution and the identification of regional disparities in care provision. Capture-recapture methods will estimate register coverage by comparing patient counts from independent data sources. Statistical analyses will be performed using SPSS (IBM Corp), Python, or MATLAB (MathWorks).

### Sites Inclusion Procedure

New sites wishing to contribute to the MND Register must complete several steps. Interested centers should contact the MND Register team via email (mndregister@kcl.ac.uk) to schedule an initial discussion. During this meeting, site representatives will be asked to provide information regarding the number of patients with MND treated at their center, the existence of a local database, contact details for their research and development department, and the geographic catchment area served. Following this, each site must submit Good Clinical Practice certification and curriculum vitae for the principal investigator and data collection personnel. Local research and development approval must be obtained, and a complete research ethics committee–approved document set must be reviewed by the local research and development team. Additionally, the site must sign a Data Sharing Agreement (Organisation Information Document) and demonstrate compliance with the GDPR, the Data Protection Security Toolkit [23], or the Welsh Information Governance Toolkit [24]. Upon completion of these requirements, the data scientist and project manager will provide training on secure data collection and transfer protocols. Data collection occurs twice annually, as described above. Once local research and development approval is granted, and all necessary training and data-sharing agreements are finalized, patient data collection can commence, officially integrating the site into the MND Register. Once a site is included in the register, they are contacted regularly every 6 months to ensure data will be shared. If any technical or practical issue is noticed or reported by the site, the MND Register team contacts the site team to support them and ensure compliance.

### Data Sharing and Linkage Procedure

The MND Register is intended as a resource for the wider research community. Data access can be requested by completing a form that is reviewed by the data access committee, which evaluates applications based on scientific merit and data requirements. The data access committee comprises chief investigators, data scientists, project managers, MND researchers, and patient representatives. The formal application must include the following information: a clear statement of the research objectives and the relevance of the requested dataset (scientific justification), a specification of the data variables necessary for the study (data fields required), and an overview of the proposed analytical approach, including methodology and expected outcomes (planned statistical analysis and publication plan; Multimedia Appendix 1). Approved researchers may then access the datasets through the KCL TRE, subject to appropriate approvals.

The MND Register is also designed to facilitate data linkage with other databases or studies to enhance research capabilities. Minimal patient data, including NHS number, date of birth, and postcode, may be securely shared with designated health organizations to enable linkage with their existing records. However, these organizations will only have access to this minimal dataset and not the comprehensive MND Register dataset. All data linkage will take place within a highly secure TRE, hosted on secure servers, where only authorized personnel can access the data under strict governance and approval processes.

### Dissemination of Findings

Project findings will be disseminated through publication in peer-reviewed scientific journals and presentation at national and international conferences. To enhance knowledge translation, key outputs will be adapted for dissemination via the MND Register, the UK MND Research Institute website, and associated digital platforms, ensuring accessibility for patients, carers, and the wider public. Engagement with research funders and policymakers will further ensure that the findings inform evidence-based policy development and strategic decision-making in motor neuron disease research and care.

### Ethical Considerations

The MND Register has undergone ethical review by the London-South East Research Ethics Committee (reference: 25/LO/0371) and has been in operation since 2015. Since 2020, it has also been covered under Section 251 of the NHS Act (2006) [25], allowing authorized health care professionals to collect patient data without individual consent in England and Wales (Multimedia Appendix 2). This was necessary to ensure that the MND Register could meet its primary objective of estimating the incidence and prevalence of MND across England and Wales. In Northern Ireland, identifiable patient data are not transferred to the MND Register; instead, only deidentified clinical and demographic data are shared. As the register functions as a research database rather than an interventional study, a Health Research Authority review was not required, and no Health Research Authority approval letter or amendment tool has been issued.

## Results

### Overview

The Motor Neurone Disease Association funds the MND Register for England, Wales, and Northern Ireland through a Healthcare Research Award that commenced on January 1, 2026, and will provide support for a period of 60 months. Data collection for the Register commenced in 2015 at King's College Hospital, with additional participating sites joining subsequently as the Register expanded nationwide. At the time of this publication, ethical and governance approvals are in place to permit continued data collection until June 2030. As a national disease register, the long-term objective of the Register team is to maintain ongoing data collection beyond this period and to seek renewed approval from the relevant Research Ethics Committee and Confidentiality Advisory Group at the end of each 5-year approval cycle. Although the Register data are not analyzed routinely by the core team, curated datasets are made available to external researchers for approved analyses, including conference presentations, posters, and peer-reviewed manuscripts. We anticipate the publication of approximately 1 manuscript per year addressing key topics such as incidence and prevalence [9], mortality, epidemiological investigations, the influence of geographic variation across the United Kingdom, and the impact of distance from specialist MND centers on clinical outcomes. Additional projects will be undertaken in collaboration with research institutions and external investigators who submit data requests and work jointly with the register team. All publications arising from the Register will be made available on the MND Register website [17] under the “Research Papers” section.

### Sites Included in the MND Register

**Figure 3.**
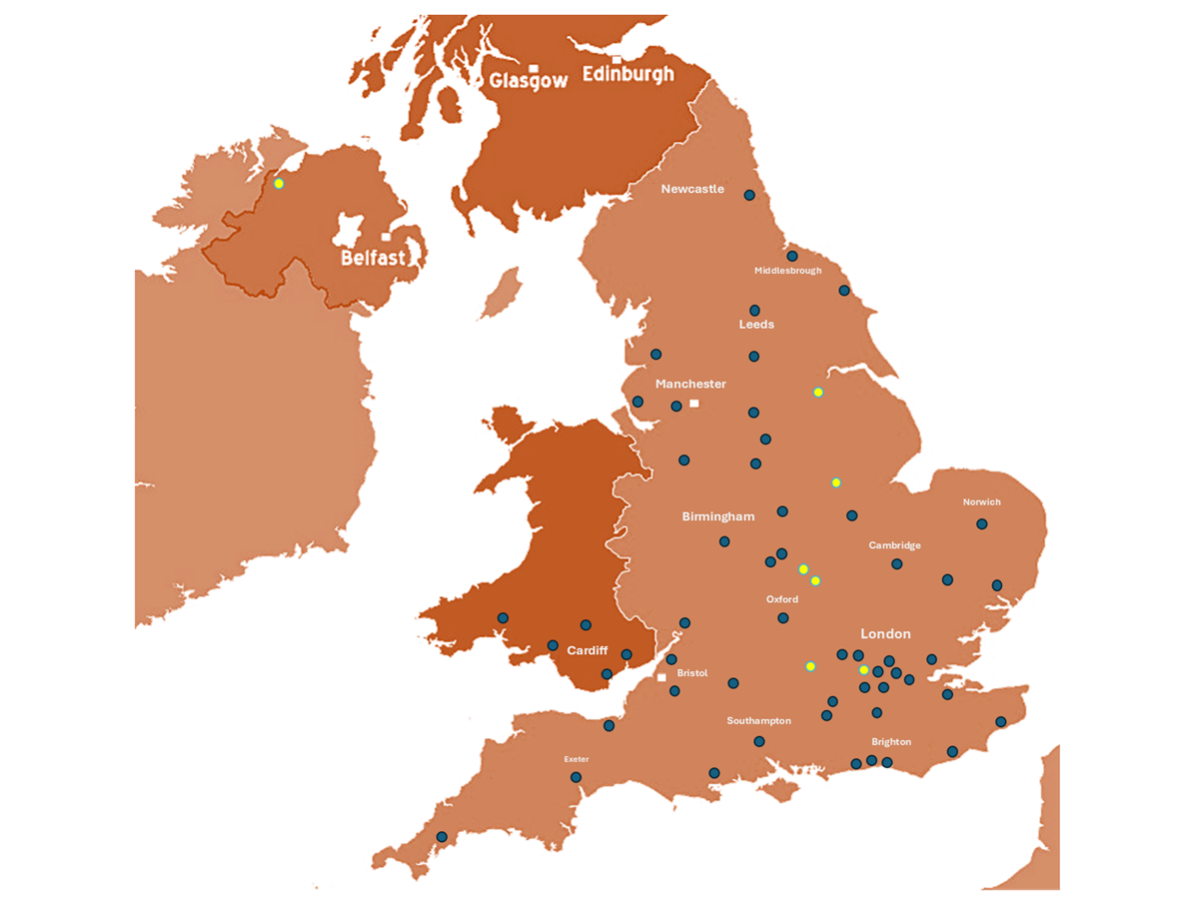
Map of sites included in the Motor Neuron Disease (MND) Register. Locations of participating sites across England, Wales, and Northern Ireland are shown. Blue markers indicate sites currently participating in the MND Register (active or paused due to lack of capacity), while yellow markers represent sites in the setup phase that are undergoing approvals or preparing to begin data collection. An updated list of sites and map will be available on the MND Register webpage [17] and updated every 6 months.

As of October 2025, a total of 60 sites (50 active, 3 active but temporarily out of capacity, and 7 setting up) are participating in the MND Register of England, Wales, and Northern Ireland (Figure 3), with each categorized by participation status (active in blue or setting up in yellow).

The “Active” category includes sites currently collecting and sharing data. The “Set-Up in Progress” group consists of sites undergoing Information Governance reviews, R&D document reviews, or waiting for principal investigator allocation. The “Awaiting Data Collection Method” status refers to sites still deciding whether to use Microsoft Access, REDCap, or Microsoft Excel for data collection. Finally, the “No Capacity” group includes sites unable to participate due to staffing shortages; these sites are regularly contacted to reassess their status.

### Data Included in the MND Register

As of April 2025, the raw register comprises data on 11,202 patients diagnosed with MND across the United Kingdom. The register includes 6948 individuals who are alive (representing over 60% of all registered cases). Among these, postcode data are available for 4341 individuals, distributed as follows: Northern Ireland (n=1), Wales (n=121), and England (n=4219). The sex distribution among living individuals is 59.1% male and 40.9% female. Anonymized demographic and clinical information for patients included in the MND Register will be publicly accessible via published scientific publications and the MND Register website [17], which will be updated annually.

## Discussion

### Key Findings and Implications for Research

We have established one of the most extensive national registries for MND globally, covering the population diagnosed with ALS in England, Wales, and Northern Ireland.

The structure of the protocol provided here aims to support the establishment of similar national registers in other countries or research centers, particularly in countries where multiple distinct bodies control health access and data. By detailing the methodology for patient identification, data governance, and secure data sharing, we hope to provide a valuable framework for others facing the challenge of developing MND registries. The strategies presented, including data integration, ethical compliance, and remote data collection methods, can guide global efforts to harmonize MND research. Beyond national efforts, the MND Register may serve as a model for international replication, supporting the harmonization of research methodologies and fostering data sharing across borders. As it continues to grow, the register is positioned to become a central hub for national ALS research and contribute to international programs of work, promoting large-scale collaborations and contributing to a deeper understanding of disease heterogeneity and progression.

The development of a structured register that supports the standardized and systematic collection of clinical and demographic data across multiple sites may then enable longitudinal monitoring, centralized and secure data storage, and provide a robust platform for high-quality data analysis. These capabilities form the basis for the production of peer-reviewed scientific publications and the dissemination of findings to both the academic community and the general public. Regular data capture across participating centers enhances the quality and consistency of the dataset, supporting a wide range of research questions.

Crucially, the register may also act as a national resource that facilitates linkage with other datasets and enables data sharing with external researchers under secure governance frameworks. The MND Register also operates within the broader UK rare disease research ecosystem and works closely with organizations such as the UK MND Research Institute and the MND Association. Integration with national datasets, including NHS Digital (Hospital Episode Statistics and Office for National Statistics mortality data), further supports collaborative research and large-scale epidemiological analyses. The register also collaborates with national research infrastructures such as Dementias Platform UK to facilitate secure access to large-scale health data. Integrating national disease registries with existing research infrastructures, patient organizations, and national health data providers may help maximize their scientific value and strengthen rare disease research. This interconnected structure strengthens collaborations, maximizes the use of collected data, and supports integrative research strategies, including the combination of clinical, genetic, environmental, and patient-reported outcomes. Looking ahead, future registers should also focus on scalability, operational efficiency, and increased patient engagement. Potential innovations include digital platforms for patient self-reporting through online surveys, which will reduce the burden on clinical teams while enhancing data granularity and timeliness. A major focus will be the integration of artificial intelligence and machine learning, which will enable more sophisticated real-time analysis of complex datasets, uncover novel insights, and improve predictive modeling. Incorporating electronic health records and natural language processing will further enhance data accuracy and streamline data collection, allowing for real-time entry and the extraction of critical information from unstructured clinical notes. However, challenges such as data privacy, system interoperability, and validation of artificial intelligence models must be addressed to fully realize these potential benefits.

### Limitations

The retrospective components of the MND Register introduce potential biases and limit the ability to establish causal relationships. Efforts to integrate prospective data collection, strengthen methodological rigor, and expand linkages with external datasets are ongoing, aiming to mitigate these limitations and enhance the scientific use of the register.

A further challenge is the incomplete data for certain variables, particularly those not consistently recorded across all sites or time points. For example, postcode information, which is used for geospatial analyses and linkage with external datasets, is not available for all participants due to differences in historical data collection practices and the retrospective contribution of data from some centers. Missing data may reduce statistical power, hinder subgroup analyses, and introduce potential selection bias. As the register transitions toward prospective data collection and standardized electronic platforms such as REDCap and TiM-R, data completeness is expected to improve.

Additionally, while the register currently includes a limited set of variables, some key data, such as genetic mutation information, are not universally available, which may constrain the comprehensiveness of certain analyses.

Finally, data collection relies on voluntary participation from clinical sites, which may influence geographical coverage and the completeness of data submission. To mitigate this, the MND Register team maintains regular communication with participating centers, provides training and technical support, and conducts periodic follow-ups to facilitate data submission. The register also collaborates with the Motor Neurone Disease Association and patient and public involvement groups to strengthen engagement with clinical services. Similar engagement strategies may benefit other national disease registry initiatives. Nevertheless, initiatives to improve data completeness, including enhanced site support, standardized collection protocols, incentivization of centers, and real-time data quality monitoring, are actively being pursued to address these challenges.

### Conclusions

The MND Register of England, Wales, and Northern Ireland provides a vital national resource for advancing our understanding of MND, enabling comprehensive epidemiological analysis and supporting clinical research. By capturing detailed demographic, clinical, and epidemiological data from across England, Wales, and Northern Ireland, the register aims to address regional disparities in care, inform health care policy, and enhance recruitment for clinical trials. While challenges such as missing data, the retrospective nature of some components, and the limited availability of genetic data remain, ongoing efforts to standardize data collection, improve data completeness, and incorporate prospective elements will strengthen the register’s utility. As the register evolves, it has the potential to contribute significantly to global MND research, fostering international collaborations and facilitating the development of personalized treatments for motor neuron disease.

## Supplementary material

10.2196/86458 Multimedia Appendix 1 Metadata UK Motor Neuron Disease (MND) Register.

10.2196/86458 Multimedia Appendix 2 Research ethic committee approval 2025.

10.2196/86458 Multimedia Appendix 3 Grant offer letter.

## Abbreviations

ALS: amyotrophic lateral sclerosis
CREATE: Computational Research, Engineering and Technology Environment
GDPR: General Data Protection Regulation
KCL: King’s College London
MND: motor neuron disease
NHS: National Health Service
REDCap: Research Electronic Data Capture
SQL: Structured Query Language
TiM-R: Telehealth in Motor Neuron Disease–Research
TRE: Trusted Research Environment
