# Supplementary material for: The Motor Neuron Disease Register for England, Wales, and Northern Ireland: Protocol for a Population Register
Source: JMIR Res Protoc. 2026 Jun 3;15:e86458. doi: 10.2196/86458 (PMC13234496; doi:10.2196/86458)
Supplement: Multimedia Appendix 2 [file resprot-v15-e86458-s002.pdf]

London - South East Research Ethics Committee
Health Research Authority
2 Redman Place
London
E20 1JQ

20 June 2025

Professor Ammar Al-Chalabi
King's College London
Department of Basic and Clinical Neuroscience
5 Cutcombe Road
London
SE5 9RX

Dear Professor Al-Chalabi

**Title of the Research Database:**	**MND Register for England, Wales and Northern Ireland**
**REC reference:**	**25/LO/0371**
**IRAS project ID:**	**357975**

Thank you for your letter of 18 June 2025, responding to the Committee's request for further information on the above research database and submitting revised documentation. The further information has been considered on behalf of the Committee by the Chair and Committee Member Anne Tholen.

Confirmation of ethics opinion

On behalf of the Committee, I am pleased to confirm a favourable ethical opinion of the above research database on the basis described in the application form and supporting documentation as revised.

This application was for the renewal of a Research Database application. The previous REC Reference number for this application was: 20/LO/1189

Additional conditions of approval

In addition to the standard conditions attached, ethical approval is subject to the following:

Publication of Your Research Summary

We will publish your research summary for the Research Database on the research summaries section of our website, together with your contact details, no earlier than three months from the

date of this favourable opinion letter. Where a deferral is agreed, a minimum research summary will still be published in the research summaries database. At the end of the deferral period, we will publish the full research summary.

Should you wish to provide a substitute contact point, make a request to defer, or require further information, please visit: https://www.hra.nhs.uk/planning-and-improving-research/application-summaries/research-summaries/

Duration of ethical opinion

The favourable opinion has been renewed for five years from the date of this letter provided that you comply with the standard conditions of ethical approval for Research Databases. You are advised to study the conditions carefully. The opinion may be renewed for a further period of up to five years on receipt of a fresh application. The fresh application should be made three - six months before the five years expires, to ensure continuous approval for the research database.

Research Database Renewals

This Research Database may be renewed for further periods of five years at a time by following the process described in the above paragraph.

Approved documents

The documents reviewed and approved at the meeting were:

Document	*Version*	*Date*
Other [List of Approved DSA Out MND Register]	1	28 April 2025
Other [Letter Of Support from MND Association]	1	24 April 2025
Other [Grant Amendment Confirmation Letter KCL]	1	31 December 2024
Other [Grant Amendment Confirmation Letter Oxford]	1	31 December 2024
Other [Document 2 MND Register Comprehensive Dataset]	1	24 April 2025
Other [Document 7 Terms of Reference for DAC]	1	24 April 2025
Other [Document 8 Data Access Request Form]	1	24 April 2025
Other [Document 9 Data Access Policy]	1	24 April 2025
Other [Document 12 Data Flow Diagram Template]	1	24 April 2025
Other [Document 3 MND Register Advert Template V2. 16.06.2025_tracked changes]	2	16 June 2025
Other [Document 4 MND Register Privacy notice Patients V2 16.06.2025_tracked changes]	2	16 June 2025
Other [Document 5 MND Register Privacy notice Researchers V2 16.06.2025]	2	16 June 2025
Other [Document 6 MND Register Opt Out V2_16.06.2025_tracked changes]	2	16 June 2025
Other [Document 10 Notification Flyer MND Register England and Wales V2_16.06.2025_tracked changes]	2	16 June 2025
Other [Document 11 Notification Flyer MND Register Northern Ireland V2_16.06.2025_tracked changes]	2	16 June 2025
Other [MND Register (IRAS 357975)_REC_responses_18 June 2025]	N/A	18 June 2025

Protocol for management of the database [Document 1 MND Register Protocol]	1	24 April 2025
REC Application Form [RD_Form_29042025]		29 April 2025

Research governance

Under the UK Policy Framework for Health and Social Care Research, there is no requirement for NHS research permission for the establishment of research databases in the NHS. Applications to NHS R&D offices through IRAS are not required as all NHS organisations are expected to have included management review in the process of establishing the database.

Research permission is also not required by collaborators at data collection centres (DCCs) who provide data under the terms of a supply agreement between the organisation and the database. DCCs are not research sites for the purposes of the RGF.

Database managers are advised to provide R&D offices at all DCCs with a copy of the REC application for information, together with a copy of the favourable opinion letter when available. All DCCs should be listed in Part C of the REC application.

NHS researchers undertaking specific research projects using data supplied by a database must apply for permission to R&D offices at all organisations where the research is conducted, whether or not the database has ethical approval.

Statement of compliance

The Committee is constituted in accordance with the Governance Arrangements for Research Ethics Committees and complies fully with the Standard Operating Procedures for Research Ethics Committees in the UK.

After ethics review

The standard conditions of the REC favourable opinion give detailed guidance on reporting requirements for research databases with a favourable opinion.

User Feedback

The Health Research Authority is continually striving to provide a high quality service to all applicants and sponsors. You are invited to give your view of the service you have received and the application procedure. If you wish to make your views known please complete the feedback survey available on the HRA website: http://www.hra.nhs.uk/about-the-hra/governance/quality-assurance/

HRA Learning

We are pleased to welcome researchers and research staff to our HRA Learning Events and online learning opportunities– see details at: https://www.hra.nhs.uk/planning-and-improving-research/learning/

**IRAS project ID: 357975**	**Please quote this number on all correspondence**

Yours sincerely

pp. 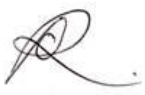

Mr Chris Foy
Chair

E-mail: londonsoutheast.rec@hra.nhs.uk

Enclosures: (RD) Conditions of Approval

Research Database – Conditions of Approval

Copy to: Prof Bashir Al-Hashimi King's College London

Confidentiality Advise Team
