# Supplementary material for: The Motor Neuron Disease Register for England, Wales, and Northern Ireland: Protocol for a Population Register
Source: JMIR Res Protoc. 2026 Jun 3;15:e86458. doi: 10.2196/86458 (PMC13234496; doi:10.2196/86458)
Supplement: Multimedia Appendix 3 [file resprot-v15-e86458-s003.pdf]

mnda motor neurone disease association

Application Number 9/2617 (Office Use)

Royal Patron
HRH The Princess Royal
Francis Crick House
6 Summerhouse Road
Moulton Park
Northampton NN3 6BJ
Tel 01604 611799
Fax 01604 624726
enquiries@mndassociation.org
www.mndassociation.org
MND Connect 0808 802 6262

Prof Ammar Al-Chalabi
Professor of Neurology and Complex Disease Genetics
Department of Basic and Clinical Neuroscience
King's College London
Institute of Psychiatry, Psychology and Neuroscience
Maurice Wohl Clinical Neuroscience Institute
5 Cutcombe Road
London
SE5 9RT

17 October 2025

Dear Prof Al-Chalabi

MND Association Research Grants
Healthcare Award

RE: The MND Register for England, Wales and Northern Ireland - Phase 2

I am pleased to advise you that the Board of Trustees of the MND Association has agreed to offer you an award of a research grant to the total value of **£856,486.72 over a 60 month** period, to start **1 January 2026**.

Summary of support offered (See original application)	1st Year	2nd Year	3rd Year	4th Year	5th Year	Total £
(a) Personal Support of Applicant	-	-	-	-	-	**-**
(b) Research Staff: No of Posts: 2 Whole Time	123,251.72	134,207.12	146,148.05	159,160.90	83,568.16	**646,335.95**
(c) Technical Staff: No of Posts: Whole/Part Time	-	-	-	-	-	**-**
Additional of Pension & NI (a) (b) (c)	35,475.52	38,762.14	42,344.40	46,248.27	24,320.44	**187,150.77**
(d) (e) (f) Expenses	3,950	3,950	3,950	3,950	3,950	**19,750**
(g) Equipment	650	650	650	650	650	**3,250**
**Total Support Offered**	**163,327.24**	**177,569.26**	**193,092.45**	**210,009.17**	**112,488.60**	**856,486.72**

The award is subject to the MND Association's conditions for grants and I enclose a copy. Please ensure you retain a copy, another (together with a copy of this letter), is for your finance department administering the grant from your side (King's College London), and a copy is to be signed by you and the appropriate person in your finance department and returned to the Association within four weeks of receipt of this letter.

By accepting this award, you are also accepting the additional conditions outlined below:

- The MND Association logo to be included on the header for the Register website.
- The opening paragraphs on both the homepage and the page about the register to state that it is ‘funded by the MND Association’.
- For the MND Association logo to be elevated from the list of logos at the bottom to occupy a line on its own under the heading of “funder”, and for this logo to link to our website.
- Use of the phrase, ‘funded by the MND Association’ in first three paragraphs of all press releases related to the Register, and for the MND Association to always be offered a quote in press releases and signoff of all press releases.
- All externally facing branding for the Register (for example, posters at conferences, pull-up banners, slides, video call backgrounds) to include the MND Association logo.
- The MND Register Steering Group membership should include a representative of the MND Association
- The grant holders commit to working with the MND Association collaboratively on projects related to communication of MND prevalence information as required during the duration of this award

It is important that:

- Once everything is in place for the project to commence, the **grant activation form** is completed and returned at least one month prior to the agreed start date (Grant Condition 10.1)

- All correspondence regarding this project is marked for the attention of Natasha Rowe, quoting reference: **Al-Chalabi/Aug25/2617-794**

- Invoices are received as soon as possible following the end of each quarter. Reimbursement is made on actual expenditure and it is essential that a **detailed** breakdown of salaries and non-pay costs incurred is included with every invoice. (Please see 'Research Grants Payment and Reporting Guidelines').

- Please include the Purchase Order Number on every invoice: **106456**

- **Annual, interim and final reports** are received as soon as possible during and after the project is completed (Please see 'Research Grants Payment and Reporting Guidelines')

- **Publications** arising from this award in scientific journals or for public distribution will acknowledge the MND Association as funders

  You are mandated to make you peer reviewed papers arising directly from this grant available through open access. These research papers should be available within the Europe PubMed Central repository as soon as possible but within six months of the publication date. (See Grant Condition 17.2 and MND Association Grantee Guide to our Open Access Policy).

- **Reprints/copies of published papers** are received as soon as possible during and after the project is completed

The MND Association logo will be forwarded to you in due course for use when giving talks on this particular aspect of your work. If you would like it sooner, please contact research.grants@mndassociation.org and we will be happy to provide a copy, together with our visual identity guidelines.

With best wishes for a successful and fruitful project,

Yours sincerely

Natasha Rowe
Research Grants Co-ordinator

Enc: Grant Conditions
Research Grants Payment and Reporting Guidelines
Grant Activation Form
MND Association Guide to our Open Access Policy
Photography Consent Form

cc Finance Department
